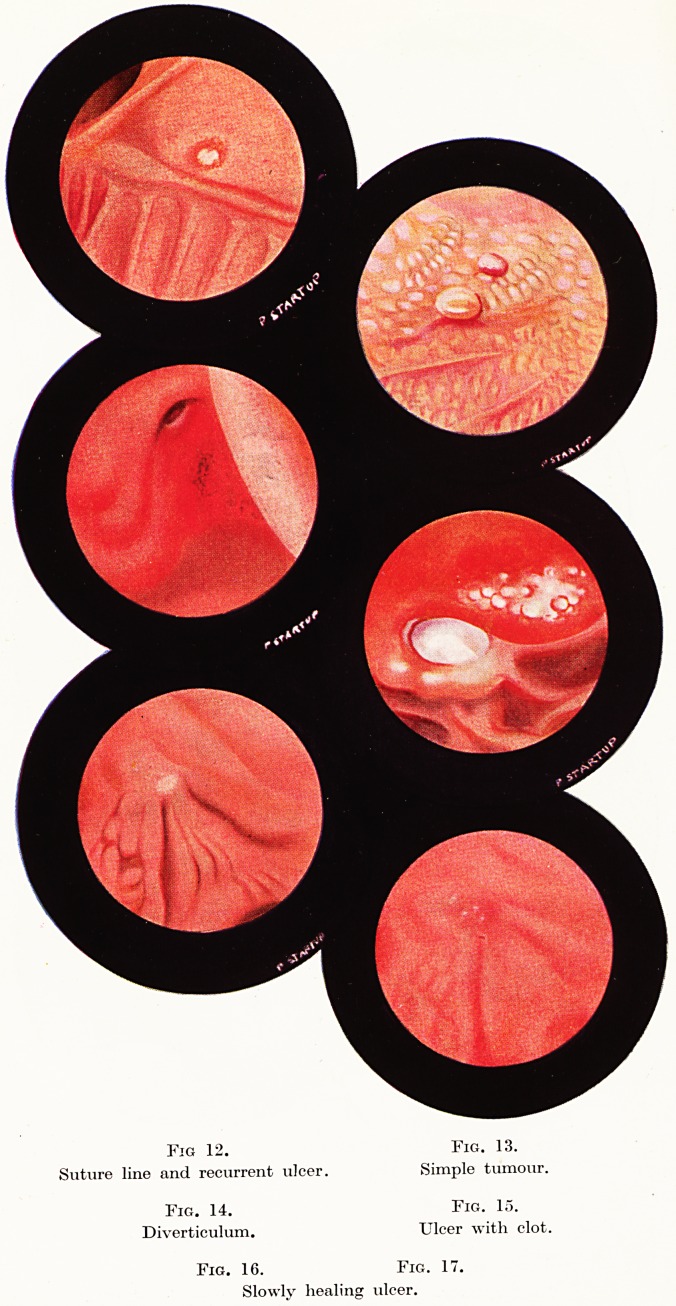# Gastroscopy: A Survey

**Published:** 1945

**Authors:** Norman C. Tanner

**Affiliations:** Senior Surgeon, St. James's Hospital, London. Surgeon Specialist to the London County Council


### The Bristol Medico-Chirurgical Journal

*"Scire est nescire, nisi id me*
*Scire alius sciret."*

AUTUMN, 1945.

### GASTROSCOPY: A SURVEY

BY

Norman C. Tanner, M.B., Ch.B., F.R.C.S.

*Senior Surgeon, St. James's Hospital, London.*
*Surgeon Specialist to the London County Council.*

Gastroscopy is the latest endoscopic method to become established by achieving safety combined with useful vision, and yet it was one of the earliest attempted. In 1868, only five years after Désormeux had invented a method of illuminating an endoscope, Kussmaul[1] published in Freiburg his attempt to examine the stomach of a professional sword swallower. His method was to pass a long straight metal tube (47cm. long and 13mm. diameter) over a previously introduced flexible obturator but the primitive method of illumination was insufficient. The first useful views of the interior of the stomach were obtained in 1881 by Mikulicz[2] using an angled rigid gastroscope with illumination at its distal end by means of a small electric bulb—then a recent innovation. This method was abandoned because of its technical difficulties and fatalities following its use. Later, attempts to make a flexible jointed gastroscope which could be made rigid and straight after introduction were made, and abandoned.

Perhaps the first routinely used gastroscope was the rigid closed tube with a long rubber tip (which facilitated passage through the lower oesophagus) introduced by Elsner[3] in 1911. Again the disadvantage was that fatalities occurred in all but one or two very

B

skilled hands, and good views could not be obtained in a large number of people : for the axis of the stomach goes forwards from the cardia and is not in the same line as the axis of the oesophagus, making rigid straight gastroscopes unsatisfactory.

THE HERMON TAYLOR FLEXIBLE GASTROSCOPE

FIG. A.

In 1932 by the co-operation of the physician Rudolf Schindler, a German now working in Chicago, and the Berlin optical instrument maker Georg Wolf, real safety was at last obtained by the invention of a flexible gastroscope which did not need to be straightened after introduction[4]. This followed the discovery that it was possible to see through a curved tube filled with many lenses each of short focal length. This instrument is rigid in its proximal half and flexible in its distal half. It has been and is widely and most usefully employed. However, it was a disadvantage that one had no control over the distal flexible half, and if it lay in close contact with the mucosa of the stomach, views of the part against which it lay could not be obtained. This difficulty was to some extent overcome when Harold Rodgers[5] introduced a rubber balloon which was fitted just above the object lens and could be inflated at will—this pushed the tip off the mucosa. In 1941 the first gastroscope with controlled flexibility was devised by Mr. Hermon Taylor with the technical assistance of Mr. R. Schranz (Fig. A). The flexible part of this instrument is shorter than is the case with the 'Wolf-Schindler,' the rigid part is longer, and the flexible part can be moved to about 40° from the straight line by a controlling wheel at the proximal end. It is gratifying that this advance has emanated from Britain but it does not represent the first experiments with gastroscopy in these islands. In 1843 Avery in England constructed an instrument through which he could get views of the upper oesophagus and about 1850 Campbell of Glasgow is said to have made a similar tube to Kussmaul's—but his sword-swallower was appalled by it and declined to perform ![7]. In 1909 Souttar and Thompson[8] constructed and used a gastroscope similar to the Mikulicz instrument, but like most such instruments it was passed under general anaesthesia and was not safe. In fact Sherren about that time declared that an exploratory laparotomy was less dangerous than gastroscopy. William Hill of London in 1911 introduced a direct vision tubular gastroscope[9] : this type not unnaturally was generally favoured by laryngologists accustomed to the oesophagoscope and it is of interest that Dr. Patrick Watson-Williams[10] of Bristol in 1911 commented on the ease with which he could see the stomach using Hill's instrument. Such views did not compare however with those we now obtain. We now know that no rigid instrument can combine the safety and adaptability of the flexible one.

The flexible gastroscope can be passed on most people of adult stature so long as there is no disease affecting the oesophagus, or spinal deformity ; the youngest I have used it on was 14, the oldest 84. The diminished mobility of the spine in the elderly is usually more than compensated for from the technical view point by their dental deficiencies. The absence of one or two teeth provides an excellent hiatus in which the rigid part of the instrument can rest, with the mouth only partially open.

The examination is done after twelve hours starvation, with the patient lying on the left side. Sensation in the mouth and pharynx is abolished by painting it with a two per cent. solution of amethocaine* hydrochloride, after which the instrument can usually be passed with only slight discomfort.

### THE ENDOSCOPIC APPEARANCE OF THE NORMAL STOMACH.

The gastroscope is passed down to the lowest part of the stomach, and finally abuts against the greater curvature. Enough air is introduced to separate the walls of the stomach and the gastroscope is rotated until the pyloric antrum is seen. At the entrance to the antrum is seen, on the lesser curvature side, an archway produced by the tangential view of the lower part of the lesser curvature—the 'angulus' roughly corresponding to the anatomical 'incisura angularis.' After a few moments peristaltic waves, which usually commence in the region of the angulus, progress towards the pylorus. As each wave reaches the pylorus this closes firmly and then its mucosa wrinkles and prolapses back into the stomach, sometimes accompanied by a squirt of duodenal juice. (Figure 1.) It is important to emphasize that if this wrinkling be not clearly seen one cannot be certain that the pylorus is normal. If the wave of contraction ends at a rigid non-prolapsing pylorus it may be the site of a carcinoma.

After a careful inspection of the pyloric antrum is completed, more air is introduced to distend the body of the stomach, and the gastroscope is slowly extracted, turning it in all directions, until the cardia is reached. Because the instrument passes down in close contact with the posterior part of the lesser curvature, it is necessary to use the controlling wheel to separate the tip from this part of the mucosa in order to view it clearly : for if it lies against the mucosa only a red glow is seen.

Along the lesser curvature four to six low longitudinal folds run from cardia to pylorus. Towards the greater curvature the rugae are thicker, more numerous and intersect more freely. Any fluid in the stomach collects in its most dependent part : with the patient lying on his left side this is the upper part of the greater curvature and is called the " mucus lake." (Figure 2.) It is green or yellowish in colour, may be clear or opaque and may have opaque mucus or saliva or sputum floating in it.

Not every part of the stomach is always seen even though the instrument be fully introduced. Indeed a realization of what one has not seen is one of the most important parts of the gastroscopist's education and one of the reasons why one always insists that gastroscopy is complementary to radiological examination and not a complete substitute for it. As the line of vision is at right angles to the line of the instrument one cannot see directly ahead and so a small area of the greater curvature will not be viewed. Sometimes the lesser curvature side of the pyloric antrum between angulus and pylorus may remain invisible especially in a very J-shaped stomach. The upper part of the fundus of the stomach is rarely seen, as it is at a higher level than the cardia. Therefore a penetrating ulcer in one of these places might be missed. A carcinoma in one of these situations or " blind areas " is usually seen because it tends to project into the stomach.

The gastric mucosa is seen to glisten with a thin film of clear mucus on its surface, and one may also see frothy material, which appears white from a distance. The colour of the mucous membrane varies from a dark congested red to pale, depending to some extent on the presence or absence of anaemia. Close inspection shews that the mucosa of different individuals exhibits variations. In the majority it is seen to be divided into multiple tiny polyhedral areas, the areae gastricae, giving in well marked cases an appearance like a pebble beach (Figure 3), but in others it is smooth and flat with no such visible divisions. Mucosa of the latter type is thinner and in extreme cases it may be so atrophic that blood vessels can be seen through it—an abnormal finding. (Figure 4.) Great distension of the stomach with air will increase the appearance of thinning. It is usually found that the mucosa with well marked *areae gastricae* has a high acid secretion, and the thin one a low secretion or achlorhydria.

### GASTRITIS.

Now we enter controversial fields. Is a very dusky red stomach inflamed ? Is opaque mucus pathological or physiological exudate ?

FIG. 1.
Normal Stomach.

FIG. 2.

The "Mucus lake."

FIG. 3.

Areae Gastricae.

FIG. 4.

Atrophic Mucosa.

FIG. 5.

Healing Ulcer.

FIG. 6.

Healed Ulcer.

FIG. 7.

Recurrent Ulcer.

FIG. 8.

Ulcer plus thrombosis.

FIG. 9.

Healing ulcer.

FIG. 10.

Healing ulcer.

FIG. 11.
Cancer.

FIG 12.
Suture line and recurrent ulcer.

FIG. 13.

Simple tumour.

FIG. 14.

Diverticulum.

FIG. 15.

Ulcer with clot.

FIG. 16. FIG. 17.
Slowly healing ulcer.

Are very large *areae gastricae* evidence of hypertrophic gastritis, or a normal type ? Is a thin mucosa merely the result of an excessive introduction of air and over distension of the stomach ? One of the first reactions to the new field of gastroscopy was to find disease in all these variations.

It is useful in this connection to hark back to some of the observations made by the U.S. Army Surgeon, William Beaumont[11], between 1822 and 1833. In 1822 in Michigan an eighteen year old French Canadian voyageur, Alexis St. Martin, accidentally sustained a severe abdominothoracic gunshot wound with perforation of the stomach. Beaumont attended to him and he survived with a gastric fistula which did not leak unduly because the gastric mucosa prolapsed into it and blocked it. This prolapse could be pushed aside and the interior of the stomach and cardia observed. Beaumont made excellent use of his opportunity to observe the gastric mucosa and secretions : and among numerous other observations he recorded that there were marked changes in colour and secretion after the introduction of food, after mechanical stimulation, excitement from alcohol, and from fear and anger.

More recently Wolf and Wolf[12] made day by day and minute by minute studies of their 57 year old subject 'Tom' who had his gastric mucosa exposed as a result of a gastrostomy performed for oesophageal stricture at the age of nine. They remarked on the increased redness and engorgement after food, alcohol, or even emotionally changed situations, and the fact that such changes were transitory.

Magnus and Rodgers have produced histological support for the view that prominent *areae gastricae*—the so-called 'pebble-beach' appearance—is an anatomical and not a pathological occurrence.

These facts help one to mark the boundary between the normal mucosa and gastritis. Certain changes in the mucosa are generally accepted as evidence of gastritis—gross irregularity of the *areae gastricae* suggesting mucosal scarring, adherent patches of opaque mucus, irregular patches of congestion, irregular patches of atrophy. With these changes may be associated erosions, polypoidal changes, or small ulcers. There appears to be a tendency for atrophic changes to appear as a terminal stage of gastritis. There is also a tendency for thinning of the mucosa to run *pari passu* with a lowering of gastric acidity with advancing years ; this is probably a degenerative change.

### GASTRIC ULCERATION.

The observation of gastric ulceration is one of the most important functions of gastroscopy. The innocence or malignancy of an ulcer is usually evident and in cases of doubt re-examination after two to three weeks of medical treatment usually dispels the doubt. Healing of the innocent ulcer by contracture and filling in of the base and ingrowth of epithelium from the edge is well seen : and one's first impression is of the strong tendency to heal, particularly in the young and in those ulcers of short duration. (Figures 5 and 6). The second impression is of the great length of time often required, after the ulcer is about five sixths healed, for the last sixth to heal soundly particularly in the aged, in chronic deep ulcers, and in those with a thinner mucosa. (Figures 8, 9 and 10, 16 and 17.) This function of shewing final healing is a most important one : and here gastroscopy is superior to radiology, for it often shews incomplete healing long after the radiological niche has disappeared. There is no doubt that many of the failures of medical treatment are due to the fact that the ulcer was never completely healed. The importance of a good healing the first time the ulcer is treated cannot be overestimated, for each breakdown results in wider scarring and induration requiring longer and longer courses of treatment : and the longer an ulcer takes to heal the greater gastric deformity results and the more likely it is to break down again, usually at the site of the previous scar. (Figure 7.)

Gastroscopy is the most efficient means of assessing different methods of treatment of gastric ulceration. Abolition of symptoms is a poor guide in a disease so subject to spontaneous remission : such remissions nearly always occur at the time of the patient entering hospital, before any specific treatment is given : in fact many patients believe the gastroscope is a form of treatment because they rarely have much pain after the first three days in hospital during which they have gastroscopy, x-ray and fractional test meal ! It is interesting to record that there is practically no difference in the rate of healing between patients in bed on special ulcer diets and frequent feeding, and patients in bed having three normal hospital meals a day (a plain and non-irritant diet it must be conceded). Similarly gastric ulcers appear to heal as well without as with alkalization. I have also observed healing to proceed normally in agitated patients, anxious about losing their jobs, and in heavy smokers. I advise my patients to abstain from smoking during treatment, but two years ago when two patients found they could not give it up I put them in a side ward and requested them to smoke as much as they possibly could. Such is human nature that one of them immediately was anxious to stop for the first time : but they smoked one about 15 the other 35 cigarettes daily, and both continued to heal though perhaps rather more slowly than usual. The latter has since relapsed and had a gastrectomy. Such findings of course are no argument against advising frequent small meals as a prophylaxis against recurrence, but suggest that continued bed rest is the major factor concerned with ulcer healing.

### GASTRIC HAEMORRHAGE.

In an ulcer which is in danger of bleeding or after a haemorrhage one may see one or more dark spots in the base which indicate the site of a thrombosed vessel end. (Figure 8.) Occasionally a clot or white slough at the edge of a crater shews from where the bleeding has come. (Figure 15.) Often severe haematemeses or melaena may result from a small acute type of ulcer, which tends to heal in ten to twenty days. By the time the patient is fit enough to have a careful radiological examination the crater is gone, but gastroscopy can often be undertaken three to seven days after the bleeding and so more often shews the ulcer. Gastroscopy considerably lowers the number of occasions on which diagnosis of gastrostaxis is made. On one occasion a gastric haemorrhage was found to be due to bleeding from multiple telangiectases extending from cardia to pylorus. This patient had many similar lesions on other parts of his body (Rendu, Weber, Osler disease).

### CARCINOMA OF THE STOMACH.

The opinion is often expressed that gastroscopy should be of value in getting patients with carcinoma of the stomach to the surgeon earlier. (Figure 11.) This is true but only to a limited extent, for the failure to get such cases early is due to no fault in the accuracy of radiological diagnoses but because in a large percentage of cases the patients arrive for investigation too late, or are inoperable when they first consult a physician. An attempt has been made to discover what one might designate a "cancer prone mucosa" with the object of keeping such cases under special observation. Carcinoma may be present in patients with a normal mucosa, but there is an increased proneness both for carcinoma and simple mucosal tumours to be associated with an atrophic mucosa. Gastric endoscopy occasionally does bring the carcinoma to surgery earlier, but it is more often the means of avoiding unnecessary laparotomy : in over sixty cases in the last five years (out of some 2,700 examinations) an area suspected from the radiological examination of being the seat of a neoplasm was clearly seen and found to be free of growth.

### OTHER INDICATIONS FOR GASTROSCOPY.

There are many other indications for gastroscopy ; after gastric operations the suture line and recurrent ulceration may be seen. (Figure 12.) Simple tumours (Figure 13), diverticula (Figure 14), granulomata and scars when seen give valuable information.

There is no doubt now that gastroscopy is well established and there is a natural tendency to contemplate its future, and consider the possibilities of a duodenoscope. Rovsing[13] in 1908 devised a duodenoscope but it could only be passed after laparotomy or via a low gastrostomy and so was of negligible practical value. Peroral duodenoscopy would probably demand prograde visualization and may follow the development of an even more flexible gastroscope.

### BIBLIOGRAPHY.

[1] Kussmaul, A., "Ueber Magenspiegelung," *Ber. d. Naturforsch. Geo.*, Freiburg, 1868, p. 112.

[2] Mikulicz, J., "Ueber Gastroskopie und Oesophagoskopie." *Ztrbl. Chir.*, 1881, p. 43.

[3] Elsner, H., *Die Gastroskopie*, Leipzig, G. Thieme, 1911.

[4] Schindler, R., "Ein Vollig Ungefahrliches Flexibles Gastroskop." *Munch. Med. Wo.*, 1932, vol. 79, p, 1268.

[5] Rodgers, H., "Device for Increasing Field in Gastroscopy," *Lancet*, 1936, ii. p. 438.

[6] Taylor, H., "A New Gastroscope with Controllable Flexibility," *Lancet*, 1941, ii., p. 276.

[7] Schindler, R., *Gastroscopy*, University of Chicago Press, 1937, p. 11.

[8] Souttar, H., and Thompson, T., "The Gastroscope and its Uses," *British Medical Journal*, 1909, vol. 2, p. 843.

[9] Hill, W., *On Gastroscopy and Oesophago-Gastroscopy*, John Bale, Sons & Danielsson Ltd., London, 1912.

[10] Watson-Williams, P., *British Medical Journal*, Correspondence, Nov., 1911, p.ii.

[11] Beaumont, W., *Experiments and Observations on the Gastric Juice and Physiology of Digestion*, J. P. Allan, Plattsburg, 1833.

[12] Wolf, S. and Wolf, H., "The Gastric Mucosa 'Gastritis' and Ulcer," *American Journal of Digestive Disease*, vol. 10, No. 1, 1943, p. 23.

[13] Rovsing, "Gastro-Duodenoscope and Diaphanoscopy," *Verhandl d. Deutsche Gesell f. Chir.*, 1908, vol. 37, p. 200.

## Figures and Tables

**Fig. A. f1:**
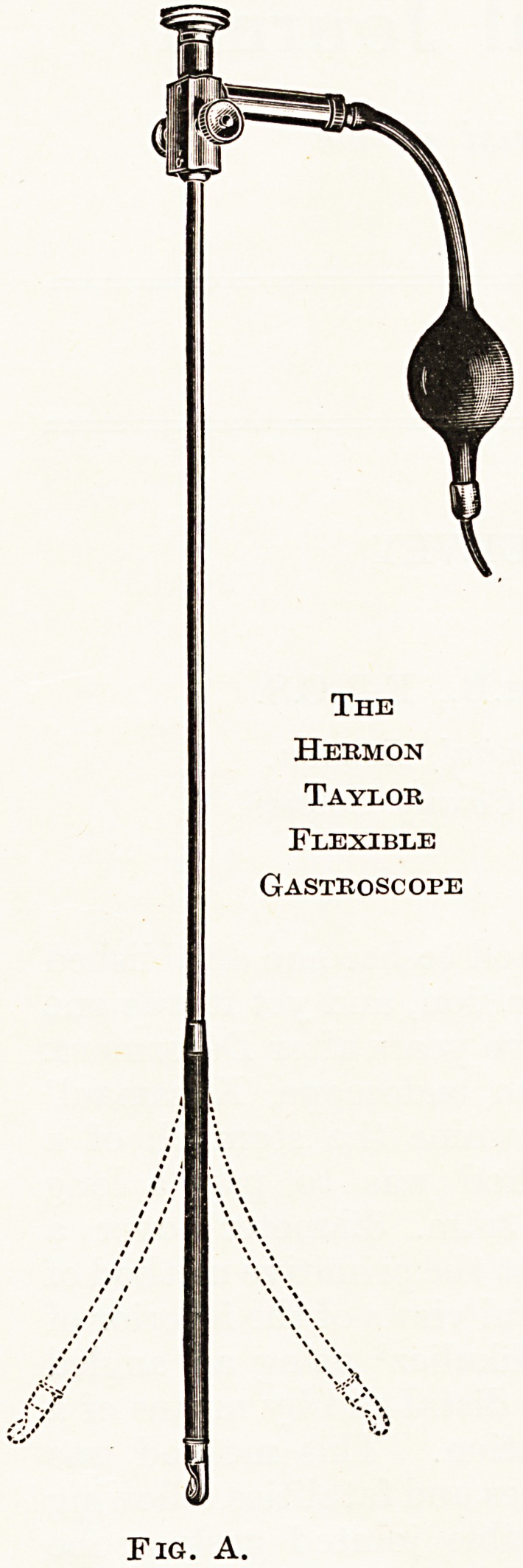


**Fig. 1. f2:**
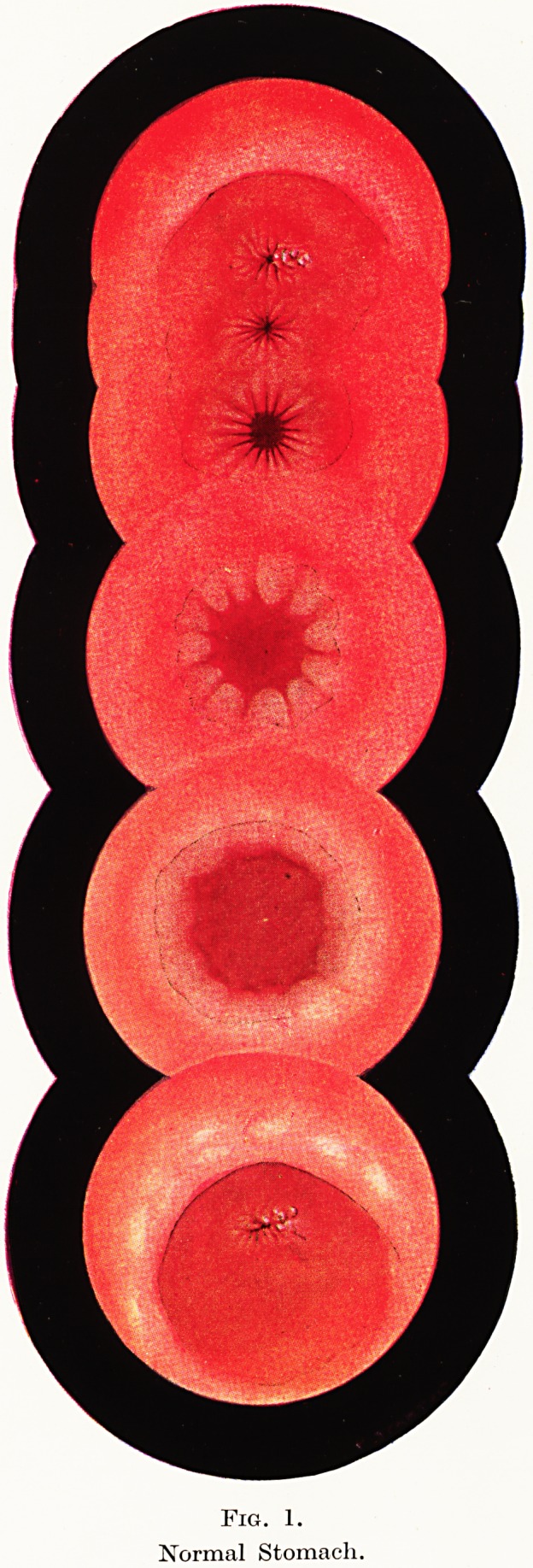


**Fig. 2. Fig. 3. Fig. 4. Fig. 5. f3:**
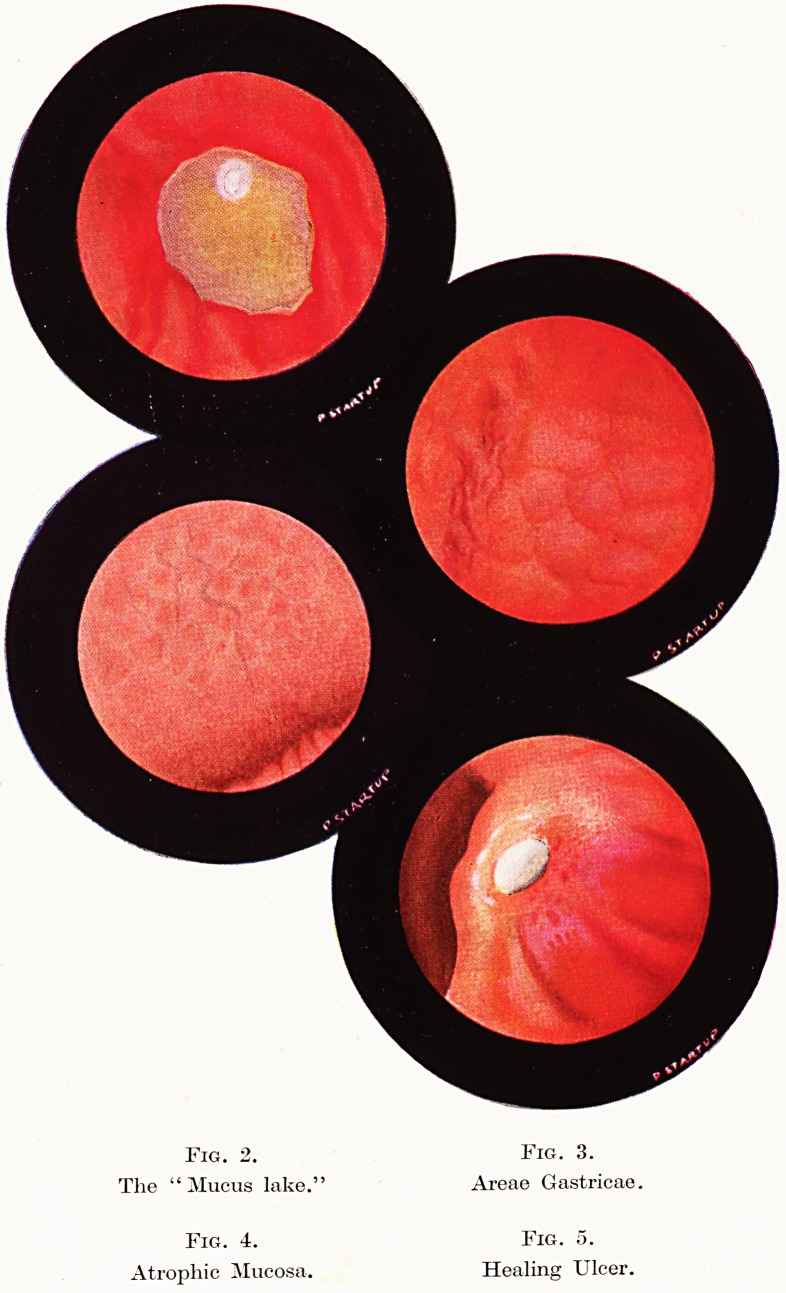


**Fig. 6. Fig. 7. Fig. 8. Fig. 9. Fig. 10. Fig. 11. f4:**
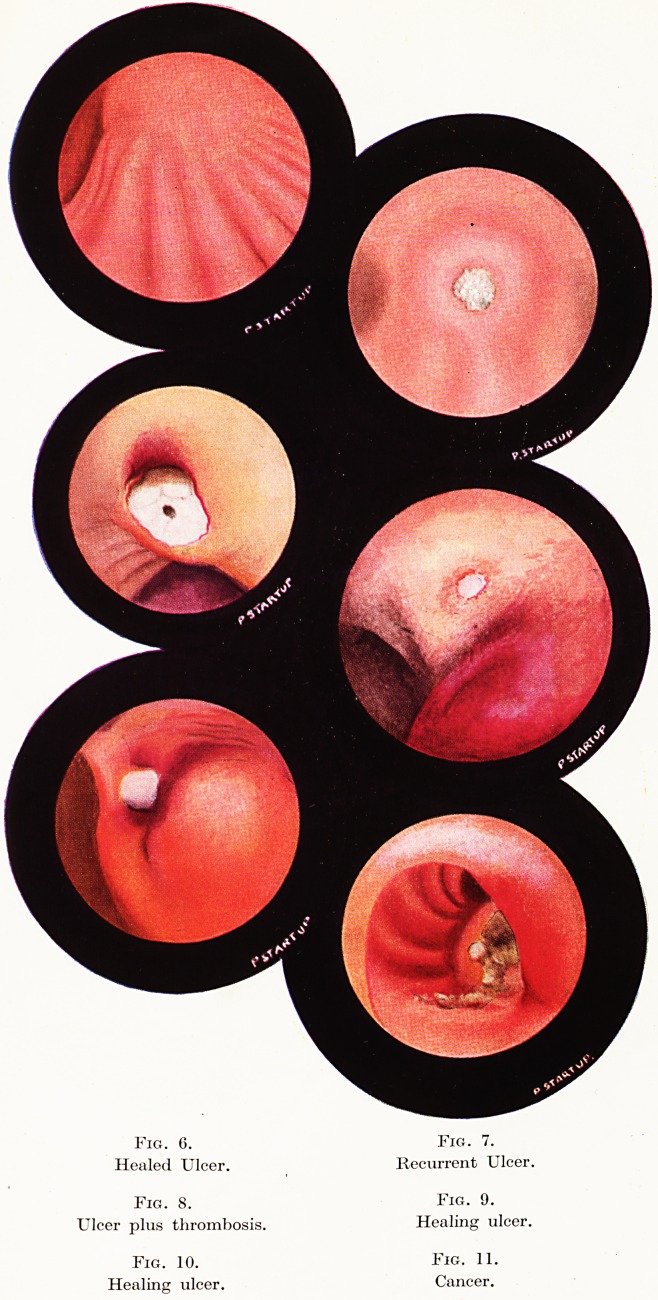


**Fig 12. Fig. 13. Fig. 14. Fig. 15. Fig. 16. Fig. 17. f5:**